# Supplementary material for: A canonical trajectory of executive function maturation from adolescence to adulthood
Source: Nat Commun. 2023 Oct 30;14:6922. doi: 10.1038/s41467-023-42540-8 (PMC10616171; doi:10.1038/s41467-023-42540-8)
Supplement: Supplementary file 1 — Supplementary Information [file 41467_2023_42540_MOESM1_ESM.pdf]

Supplementary Information

A Canonical Trajectory of Executive Function Maturation from Adolescence to Adulthood

Authors: Brenden Tervo-Clemmens[1,2,3*], Finnegan J. Calabro[4,5], Ashley C. Parr[4], Jennifer Fedor[4,6], William Foran[4], and Beatriz Luna[3,4]

Affiliations:

[1]Department of Psychiatry & Behavioral Sciences, University of Minnesota
[2]Masonic Institute for the Developing Brain, University of Minnesota
[3]Psychology, [4]Psychiatry, [5]Bioengineering,[6]Biostatistics University of Pittsburgh

Brenden Tervo-Clemmens, Ph.D.
(Corresponding Author)
btervocl@umn.edu

Supplementary Methods

Sociodemographic Characteristics of Included Datasets

By design based on different sampling procedures (see Methods) for independent datasets, sociodemographic characteristics varied across samples (Supplementary Table S1). Studies were however, balanced for biological sex at birth and in the aggregate, met and exceeded diversity in national patterns (cf., American Community Survey, Table S1) of race and ethnicity. Through a series of sensitivity analyses we demonstrated that primary results were consistent across males and females, unchanged when covarying for socioeconomic indicators and assessments of culturally acquired knowledge (Supplementary S13-S17; see also below Supplementary Methods, Sensitivity Analyses). Following a suggestion from a reviewer we also explored population weighting to match estimates from the American Community Survey, with methods adapted from those developed for a recent, large-scale multi-site developmental approach[97]. Inverse propensity weighting was used to match the characteristics of race/ethnicity, biological sex at birth, and where available, family income, of two datasets (NKI, PNC; we note these datasets were chosen given their large sample sizes and broader representation of diverse groups) to those from the ACS 2011-2015. Design-based analyses utilizing these weights and spline-based modeling (via the *survey* package in R) returned nearly identical results to those reported in the primary analyses (Supplementary Figure S2). The generalizability of the primary results here is further supported by independent replication across the included datasets.

Sensitivity Analyses

A series of sensitivity analyses we performed to examine the robustness of the primary results of the maturational timing of executive function, using domain general composite accuracy and latency measures. First, we stratified results by sex (which was self-reported in all four datasets), comparing males versus females (Supplementary Figure S13). We also determined that the results were unchanged when i) covarying for self-reported parental/guardian education (Supplementary Figure S14), ii) self-reported family income (Supplementary Figure S15), assessed via self-report and coded according to the structure likewise used in Supplementary Table S1, and iii) culturally acquired knowledge for datasets where available (Supplementary Figure S16), assessed via verbal reasoning (NCANDA, NKI, PNC datasets with the Penn CNP Verbal Reasoning Test [number of correct responses])) and vocabulary (NCANDA: Penn Vocabulary Test Correct Response [number of correct responses]; NKI WASI-II Vocabulary [T score]). Finally, in the three datasets (NCANDA, NKI, PNC) that as community samples, did not exclude participants on the basis of mental health presentations, we ensured that our primary results remained unchanged remained consistent across mental health inclusion/exclusion thresholds (Supplementary S17). Here, more restrictive thresholds for mental health inclusion/exclusion were based on prior work, data availability, and efforts to promote reproducibility and maximize sample size. NCANDA participants completed a structured clinical interview at each visit (Sem-Structured Assessment for the Genetics of Alcoholism [SSAGA]) and were coded as having current, primary psychiatric condition (i.e., not included in the restricted sample in the sensitivity analysis) if they met criteria for any DSM-IV or DSM-VS SAGA Diagnosis, except Nicotine Dependence. The NKI dataset includes summary information detailing whether each participant has “No Diagnosis or Condition on Axis I” and only those participants were included in restricted analysis sample. Following prior work with the PNC sample, the current sensitivity analyses excluded participants (i.e., not included in the restricted analysis sample) if they reported current psychoactive medication use or a history of psychiatric hospitalization.

Supplementary Figures

Supplementary Figure S1. Age Range and Study Design of Datasets

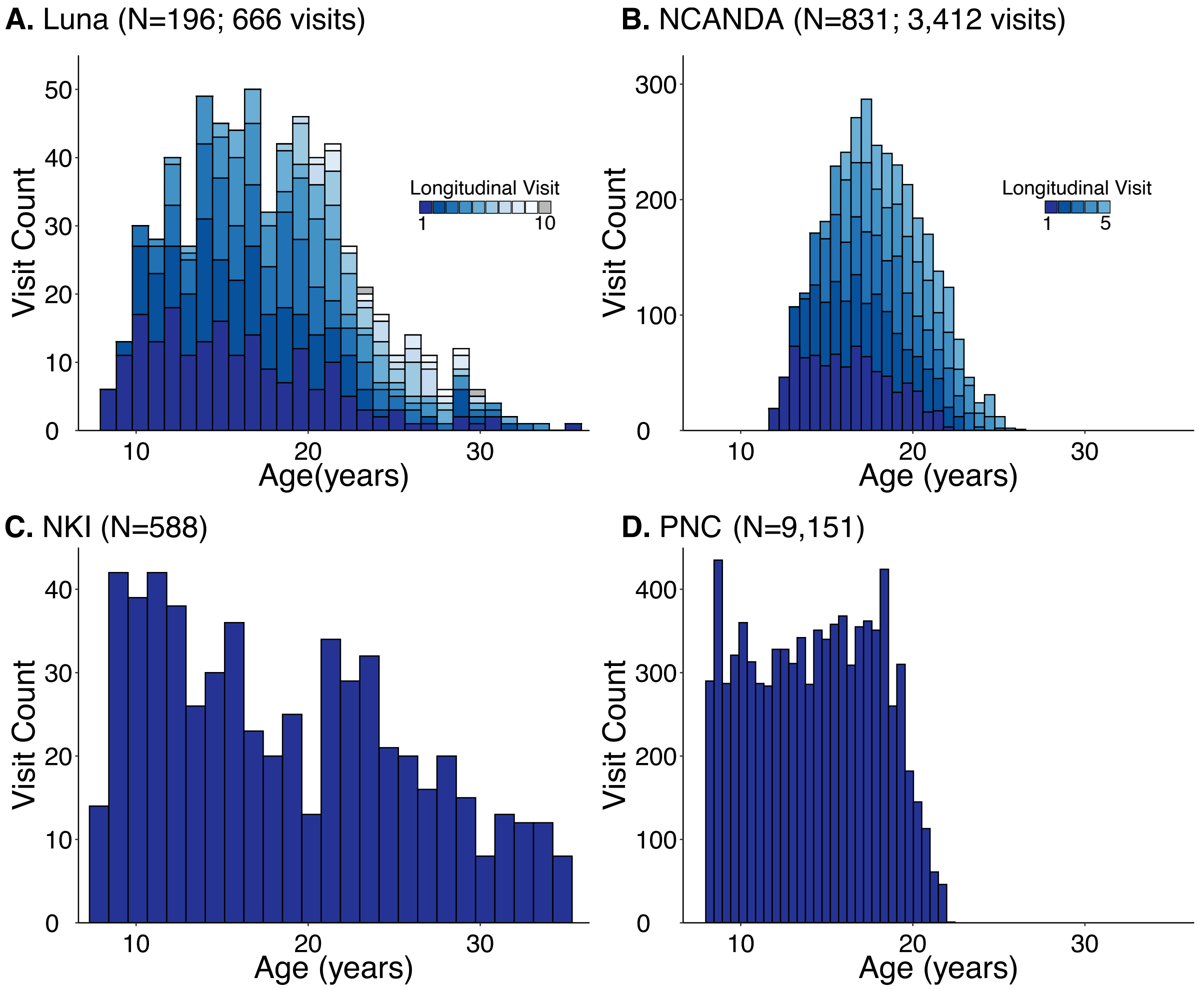

Histograms of participant age and visit number. **A)** Luna dataset (N=196; 666 total visits) used an accelerated longitudinal design with up to 10 visits per-participant. **B)** NCANDA (N=831; 3,412 total visits) used an accelerated longitudinal design with up to 5 visits per-participant. For **A, B** color indicates longitudinal visit number. Included versions of **C)** NKI (N=588) and **D)** PNC (N=9,151) studies used large cross-sectional datasets. Combined, datasets span the full adolescent period and relevant transitional periods from late childhood and early middle age adulthood (total age-range: 8-35-years-old).

Supplementary Figure S2. Executive Function Development is Consistent with Primary Results when Weighting to Match the American Community Survey

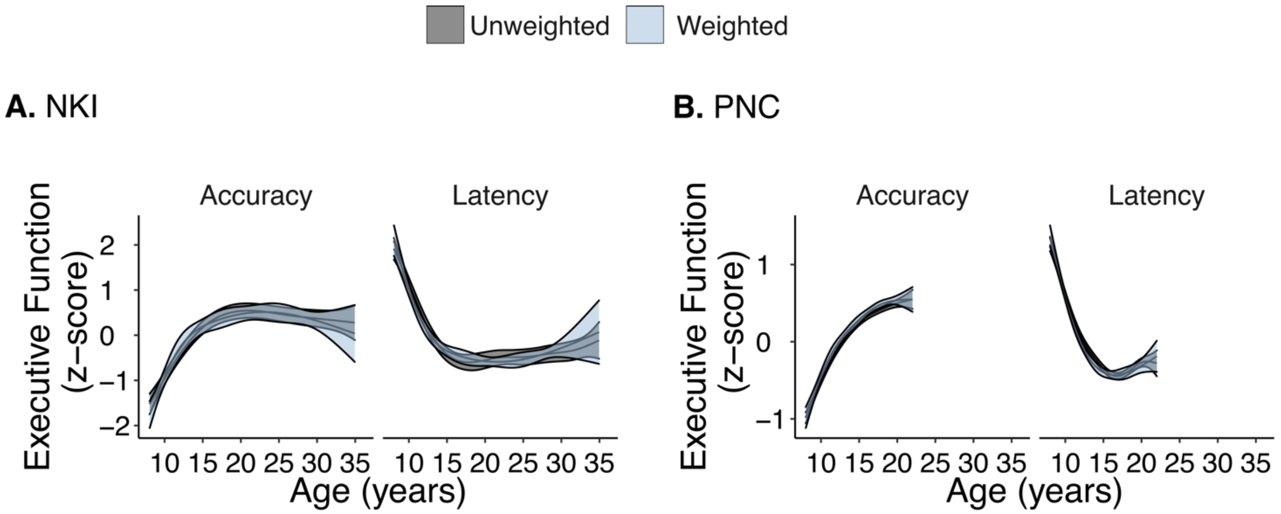

Plots display age trajectories of domain general accuracy and latency executive function measures (equally weighted composite metrics, z score sum of all accuracy, latency measures) for base GAM models for NKI and PNC datasets. Grey plots are from primary analyses used throughout the manuscript. Light blue plots are design-based analyses utilizing inverse propensity weighting to match sociodemographic characteristics of the 2011-2015 American Community Survey (see Supplementary Table S1). For NKI, the variables used in the weighting procedure were race/ethnicity, sex, age, and family income. For PNC, the variables used in weighting procedure were race/ethnicity, sex, and age.

Supplementary Figure S3. Confidence Intervals from GAM/GAMM Derivatives

A.

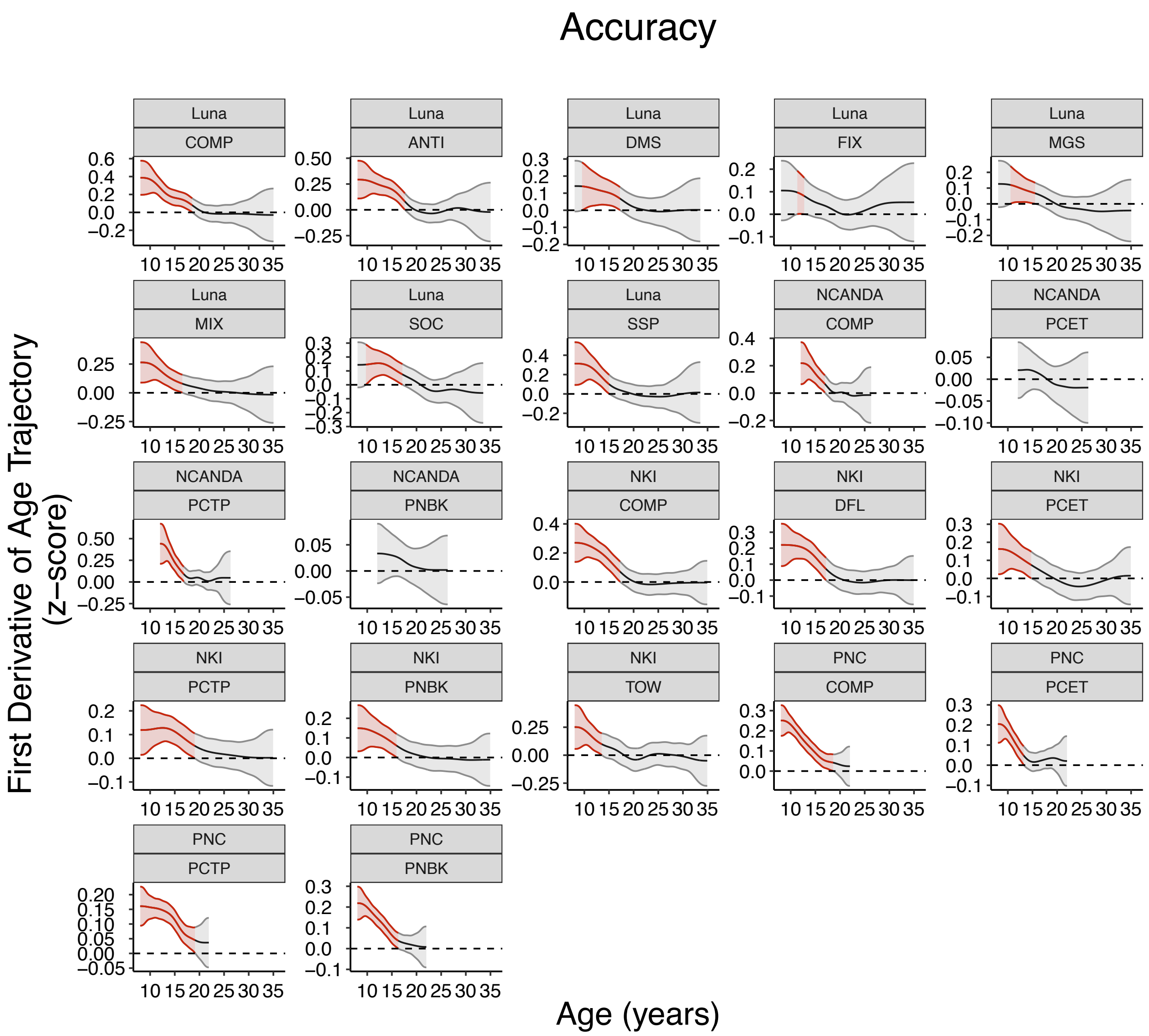

B.

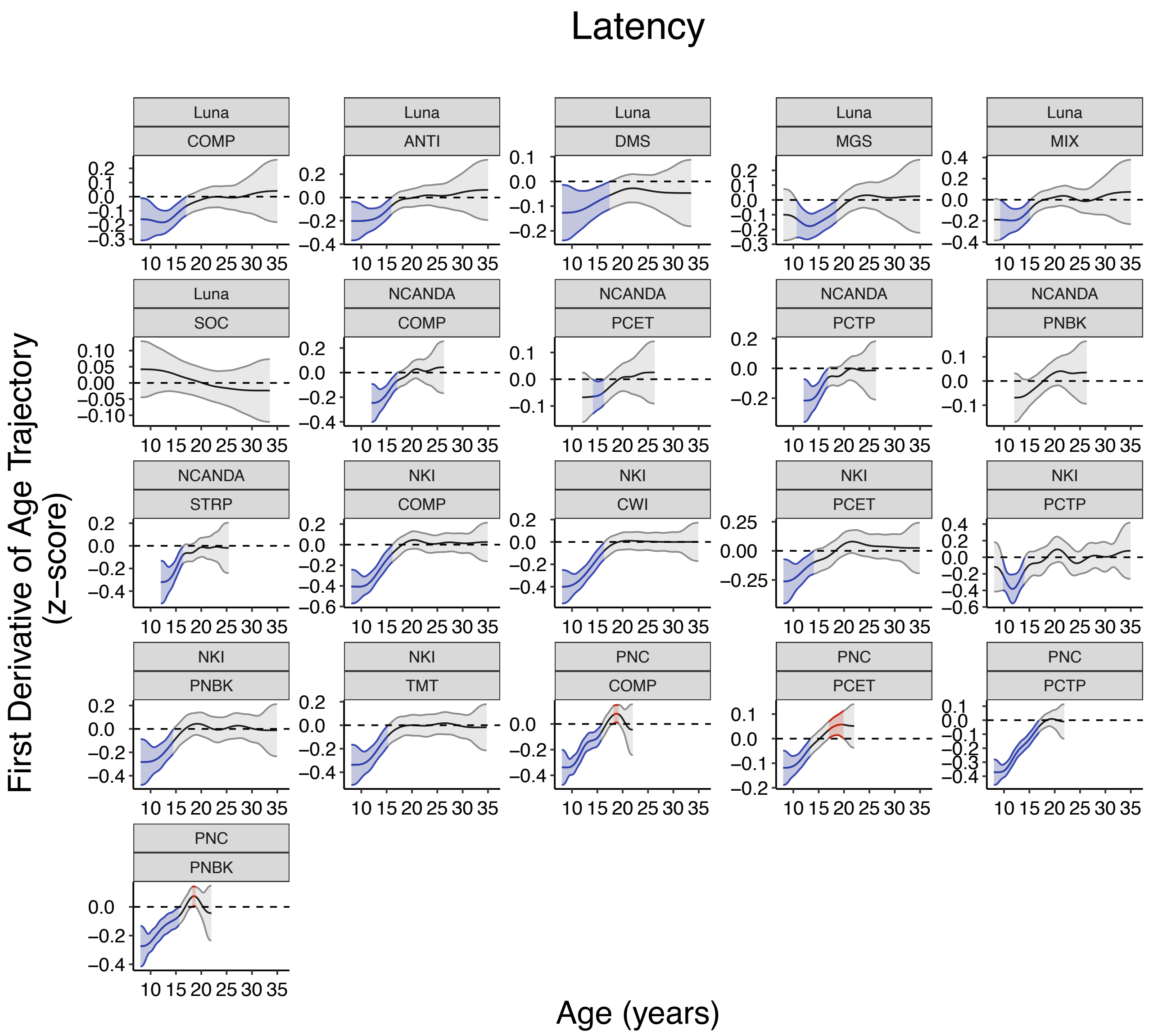

To assist in the visualization of Main Text Figure 2, plots display the first derivative (line) and its 95% confidence interval of the age trajectory of accuracy (A) and latency (B) executive function measures. Within each plot, the hashed line represents zero. The line and confidence intervals are colored (red for positive, blue for negative) if they do not include zero (e.g., $p < .05$). Main Text and Methods for details on significance testing, Main Text Figure 2 for additional visualizations.

Supplementary Figure S4. Final Age of Significant Age-Related Change (via First Derivative) for All Executive Function Measures

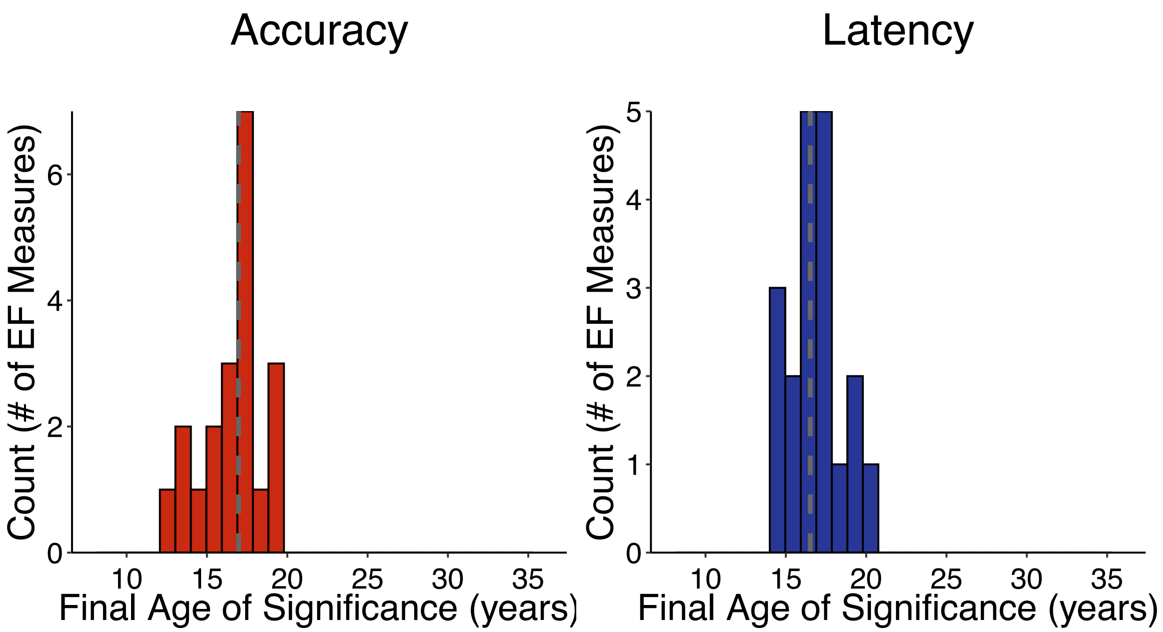

To assist in the visualization of Main Text Figure 2, histograms display the final age (in years) for all accuracy (left, red) and latency (right, blue) executive function measures. The hashed line indicates the median across measures. See Main Text and Methods for details on significance testing, Main Text Figure 2E for fully continuous aggregate analysis (three-level meta-analysis) of significant periods of age-related change.

Supplementary Figure S5. Scaled GAM/GAMM Fits Support Executive Function Maturation between 18- and 20-years-old.

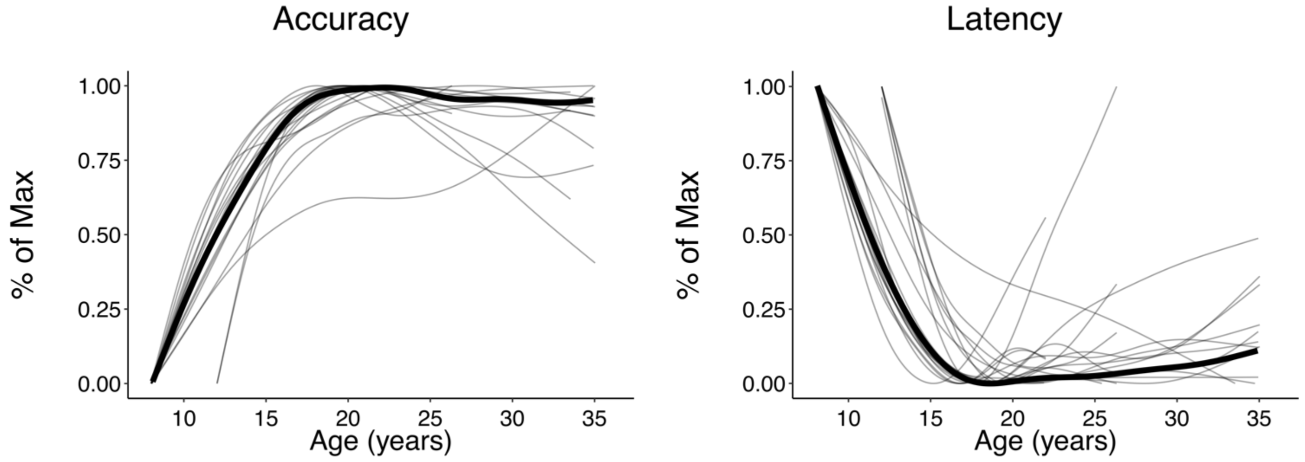

Percent of maximum GAM/GAMM fits by age for accuracy (left) and latency (right) measures from all measures from all four datasets with corrected, significant age effects. Light grey lines display individual measures. Black line displays aggregate effect (three-level meta-analysis; measures nested within tasks and datasets; smoothed for visualization). On average (meta-analytic estimate), over 95.0% of total age-related increases occurs by 18-years-old and 99.0% by 20-years-old for accuracy and over 99.7% occurs by 18-years-old for latency. Sensitivity analysis removing the PNC dataset, which only includes participants up to 20-years-old, resulted in nearly identical estimates: over 96.1% by 18-years-old and 99.4% by 20-years-old for accuracy and 98.8% by 18-years-old and over 99.9% by 20-years-old for latency.

Supplementary Figure S6. No Systematic Evidence that the Total Executive Function Variance Explained by a Single Domain General Factor Varies by Age.

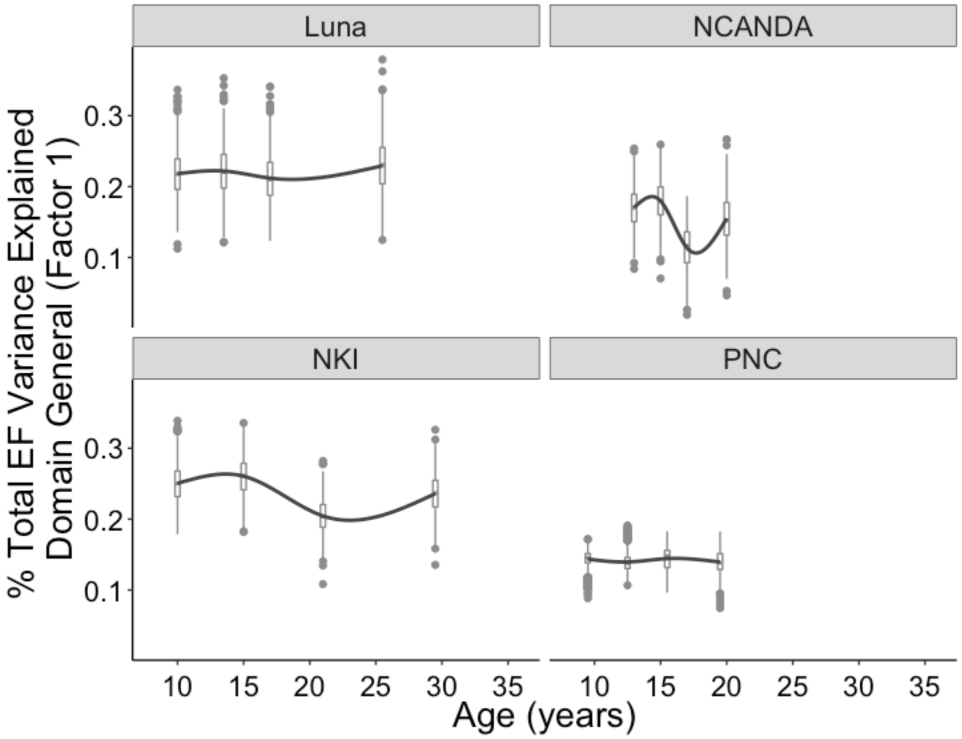

Total executive function (EF) variance explained by a domain general executive function factor (first factor via bifactor rotation; see also Main Text Figure 3) as a function of participant age. For each dataset, the domain general executive function factor was extracted for each of four independent equally sized age bins (quartiles). Boxplots (center line, median; box limits, upper and lower quartiles; whiskers, 1.5x interquartile range; points, outliers) display bootstrapped distributions of repeating this process via participant resampling within each bin 1,000 times with replacement. Boxplots are displayed on x-axis according to the midpoint age in years of the quartile bin. GAM models were used to generate lines connecting boxplots for visualization. For consistency across datasets, data presented here are from cross-sectional samples (i.e., the first visit from Luna and NCANDA data; see also Main Text Figure 3, Supplementary S9 for additional details on longitudinal data).

Supplementary Figure S7. Factor Analytic Results from Individual Datasets.

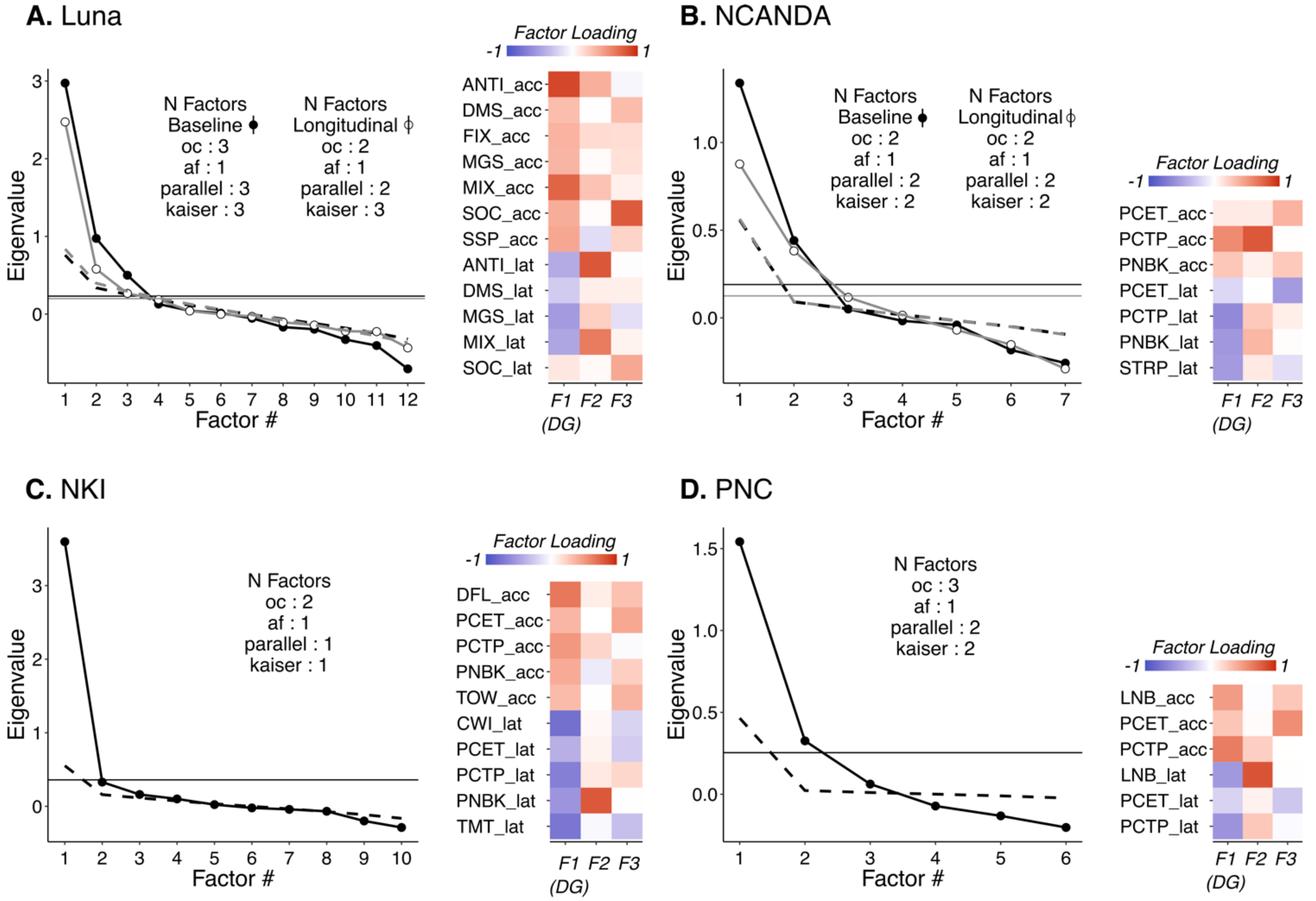

Factor analytic results for each dataset. Left figure in each panel displays eigenvalues from extracted factors (maximum likelihood method via *parallel* function in *psych* R package[82]). Hashed line displays null result from simulated, random normal data in parallel analysis (used in parallel analysis threshold for factor inclusion: "parallel" label within inset text boxes). Solid grey line (dark grey for baseline [Luna, NCANDA] and cross-sectional datasets [NKI, PNC]; light grey for longitudinal components [Luna, NCANDA]) displays the mean eigenvalue across all factors (used for factor analytic Kaiser rule). Additional data-driven thresholds (all derived from *nScree* function in *nFactors* R package[83]) include optimal coordinate (oc) and acceleration factor (af). The values from all data-driven thresholds across datasets are displayed in aggregate in Main Text Figure 3B. Right figure in each plot displays factor loadings for the first three factors with bifactor rotation. The first factor displays loadings for domain general (DG) executive function. For datasets with longitudinal data (Luna, NCANDA), these factor loadings are from cross-sectional (baseline) components. See also Main Text Figure 3C for loadings of first factor for longitudinal and cross-sectional data.

Supplementary Figure S8. Workflow for Domain-General Versus Measure Specific Age-Related Executive Function Differences.

1. For each dataset, for each measure, fit three GAM models and extract percent deviance explained.

    A. Age ~ s(measure$_{x_i}$) → Percent Deviance Explained

    B. Age ~ s(composite metric$_{M \not\ni x}$) → Percent Deviance Explained

    C. Age ~ s(measure$_{x_i}$)+s(composite metric$_{M \not\ni x}$) → Percent Deviance Explained

2. Calculate incremental deviance explained by measure$_{x_i}$ over composite metric$_{M \not\ni x}$(from variables: set *M ;* excludes measure$_{x_i}$ and all other metrics in the same primary subdomain, see Table 1).

    Δ Deviance = Percent Deviance Explained$_{\text{model C}}$ - Percent Deviance Explained$_{\text{model B}}$

3. Scale Δ Deviance to percent of initial age-relationship for measure$_{x_i}$ (model A) to estimate percent of measure specific age-related EF. Remaining percentage set to domain-general process.

    Percent Measure Specific EF = Δ Deviance / Percent Deviance Explained$_{\text{model A}}$

    Percent Domain General EF= 1 - Percent Measure Specific EF

Model comparison was used to estimate domain-general versus measure specific contributions to age-related executive function differences. First (**1**) three GAM models were fit for each dataset for each measure assessing the relationship between age and the specific measure i from subdomain x (measure$_{x_i}$): model A, a composite metric created from all measures not in the same putative subdomain as measure$_{x_i}$: composite metric $_{M \not\ni x}$, where $_{M \not\ni x}$ represents the set (M) of executive function measures that does not contain measures from subdomain x: model B, and a model where age is estimated from both measure$_{x_i}$ and composite metric $_{M \not\ni x}$: model C. As in primary analyses, the relationship between age and each measure was modeled with penalized splines, denoted with *s(x)* in above. For each model (A-C), the percent of deviance explained in age was extracted (following standard estimation in *mgcv* GAM model). Next (**2**), the incremental deviance of age explained by measure$_{x_i}$ over composite metric $_{M \not\ni x}$ was computed. Finally (**3**), the resulting measure specific age-related deviance was scaled to the original deviance estimate for the specific measure (model A) to create a percent of the original measures age effect. The remaining percentage of the model A's deviance was assigned as the domain-general percentage. To ensure consistent interpretability of the directionality of composite metric $_{M \not\ni x}$, measures from the opposing response type were sign flipped (e.g., latency sign flipped before creating equally weighted composite with accuracy measures).

Supplementary Figure S9. Non-Developmental, Visit Effects are Evident in Longitudinal Executive Function Measures

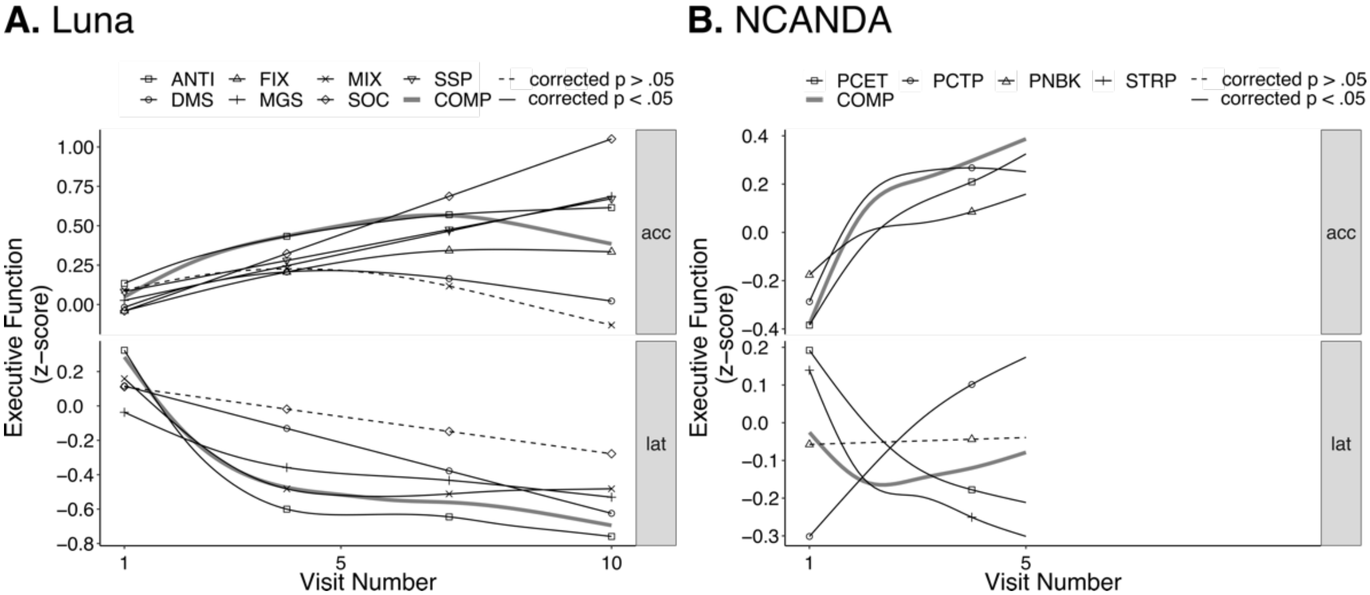

All measures scaled to per-dataset standard deviation (z) units. **A)** Non-linear fits for visit effects from the Luna dataset (N=196; 666 total visits) of general additive mixed model (GAMM; multilevel penalized spline regression) for Antisaccade (ANTI), Fixation Breaks (FIX), Mixed Antisaccade (MIX), Spatial Span (SSP), Delayed Matching to Sample (DMS), Memory Guided Saccade (MGS), Stockings of Cambridge (SOC), and equally weighted composite metrics (z score sum of all accuracy, latency measures; COMP) for accuracy measures (top) and latency (bottom). All models covaried for a smoothed effect of age. Solid line indicates models with Bonferroni corrected significance. Dashed indicates models that do not surpass this threshold. **B)** Non-linear, GAMM fits for visit effects from the NCANDA dataset (N=831; 3,412 total visits) for Penn Conditional Exclusion (PCET), Penn Continuous Performance (PCTP), Penn N Back (PNBK), and Stroop (STRP) tests and equally weighted accuracy, latency composite metrics (COMP). All models covaried for a smoothed effect of age. Solid line indicates models with Bonferroni corrected significance. Dashed indicates models that do not surpass this threshold (Luna: MIX accuracy, SOC latency; NCANDA: PNBK latency). As in previous longitudinal investigations of computerized and neuropsychological performance, age-independent visit effects (e.g., practice effects) on cognitive testing were observed for many executive function tasks. However, given that all longitudinal analyses covaried for these non-linear, smoothed effects of visit and our primary analyses and inference were additionally based on two cross-sectional, independent replication datasets (NKI, PNC), the primary results from this manuscript are judged to be robust to non-developmental visit (e.g., practice) effects.

Supplementary Figure S10. The Precision and Absolute Explanatory Power of Domain General Executive Function in Age-Related Executive Function Differences Depends on the Precision of the Composite Metric.

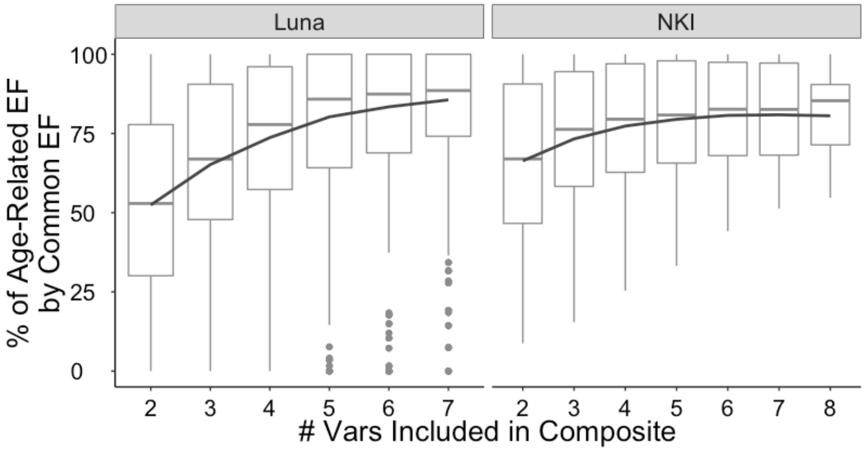

Percent of age-related differences in executive function in Luna (left) and NKI (right) datasets accounted for by domain general composite metric constructed from varying numbers of out-of-domain variables (via model comparison; see Main Text Figure 4, methods). For each dataset, all possible combinations for *n,* number of variables included in the out-of-domain composite metric (x axis), were computed and their explanatory power of age-related executive function differences (y axis) on each outcome variable was charted. Light grey boxplots (center line, median; box limits, upper and lower quartiles; whiskers, 1.5x interquartile range; points, outliers) display the results across all possible combinations of out-of-domain composite metrics and outcome metrics for each number of variables included in the composite metric. Values below zero or above one in these plots were set to zero or one respectively. Dark grey lines display smoothed mean estimates of these results.

Supplementary Figure S11. Workflow for Validation of Age Basis Function from Canonical Executive Function Trajectory.

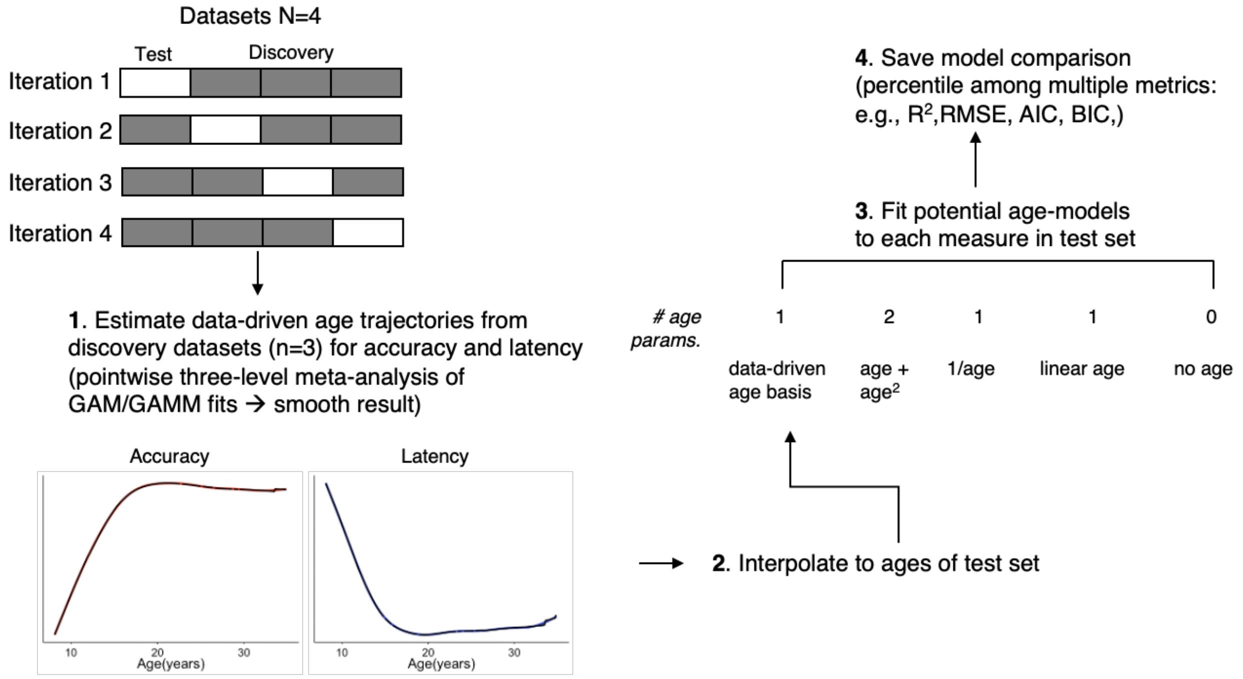

Cross-validation ("leave one dataset out") was used to validate the age basis function derived from the canonical executive trajectory. In each iteration, three (out of four) datasets were used to generate canonical executive function trajectories for accuracy and latency measures (measures aggregated across datasets via pointwise three-level meta-analysis of GAM/GAMM age fits; step **1**). The resulting output was then smoothed (via a subsequent GAM model), interpolated to the ages of the test ("left out") dataset (step **2**) and fit as a single age parameter to each accuracy and latency measure of the left-out dataset and compared to typical age models (quadratic [age+age^2], inverse age [1/age], linear age [age]) as well as an intercept only (no age) model (step **3**). Potential age models were evaluated with multiple metrics of model fit and complexity (step **4**; longitudinal models [Luna, NCANDA]: R^2, adjusted R^2, Intraclass Correlation Coefficient [ICC], Root Mean Square Error [RMSE], residual standard deviation [Sigma], Akaike's Information Criterion [AIC], Bayesian Information Criterion [BIC]); cross-sectional models [NKI, PNC)]: R^2, adjusted R^2, RMSE, Sigma, AIC, BIC).

Supplementary Figure S12. The Performance of an Out-of-Sample Basis Function Depends on Developmental Precision.

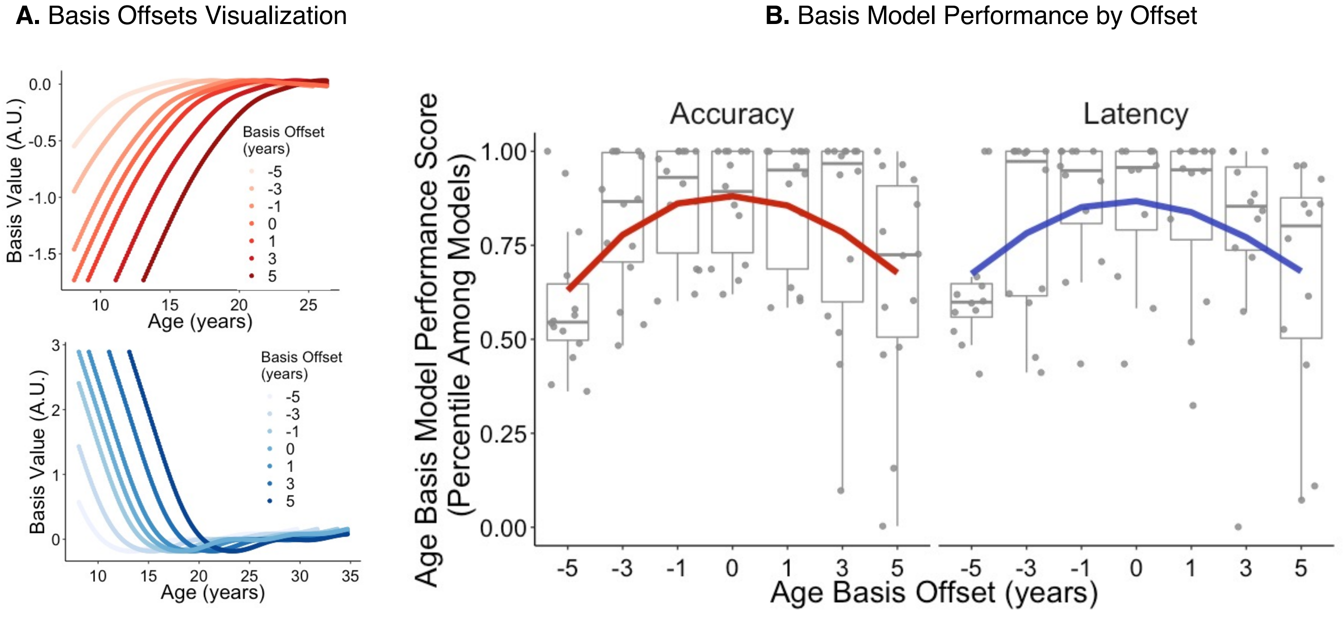

The two samples with the broadest age ranges (Luna, NKI) were used in these analyses to explore a wide range of potential offsets of the basis function (shifting basis function earlier or later with respect to years: **A**, ranging from a shift five years earlier [-5] to five years later [5] from the mean trajectory[0]). Analyses in **B.** paralleled those from Main Text Figure 5. In each iteration (Luna, NKI), one dataset was used to generate canonical executive function trajectories for accuracy and latency measures (mean of GAM/GAMM age fits). The resulting output was then smoothed (via a subsequent GAM model), interpolated to the ages of the test ("left out") dataset and fit as a single parameter (see Supplementary Figure S11 for workflow diagram of procedure) to each accuracy and latency measure of the left-out dataset. The basis function was then offset with respect to years for a range of values (**A**). Potential age models were evaluated with multiple metrics of model fit and complexity (longitudinal models [Luna]: R^2, adjusted R^2, Intraclass Correlation Coefficient [ICC], Root Mean Square Error [RMSE], residual standard deviation [Sigma], Akaike's Information Criterion [AIC], Bayesian Information Criterion [BIC]); cross-sectional models [NKI)]: R^2, adjusted R^2, RMSE, Sigma, AIC, BIC). Using the performance package (rank function) in R[55], model fit metrics were scaled 0 (worst model on that fit metric) to 1 (best model on that fit metric, accounting for the directionality of improved fit for each metric [e.g., R^2 larger values, RMSE lower values]) across candidate age models and the mean value across all model fit metrics was taken for each candidate age model to create an overall performance score. The y-axis in **B.** displays the performance score of the data-driven basis function across varying levels of offset years. Light grey boxplots (center line, median; box limits, upper and lower quartiles; whiskers, 1.5x interquartile range; points, outliers) display all performance scores across all measures from Luna and NIK datasets (N=14 accuracy; N=12 latency). Red (accuracy) and blue (latency) lines display smoothed mean estimates (via GAM model) to these results.

Supplementary Figure S13. The Magnitude and Timing of Executive Function Development is Consistent with Primary Results Across Females and Males

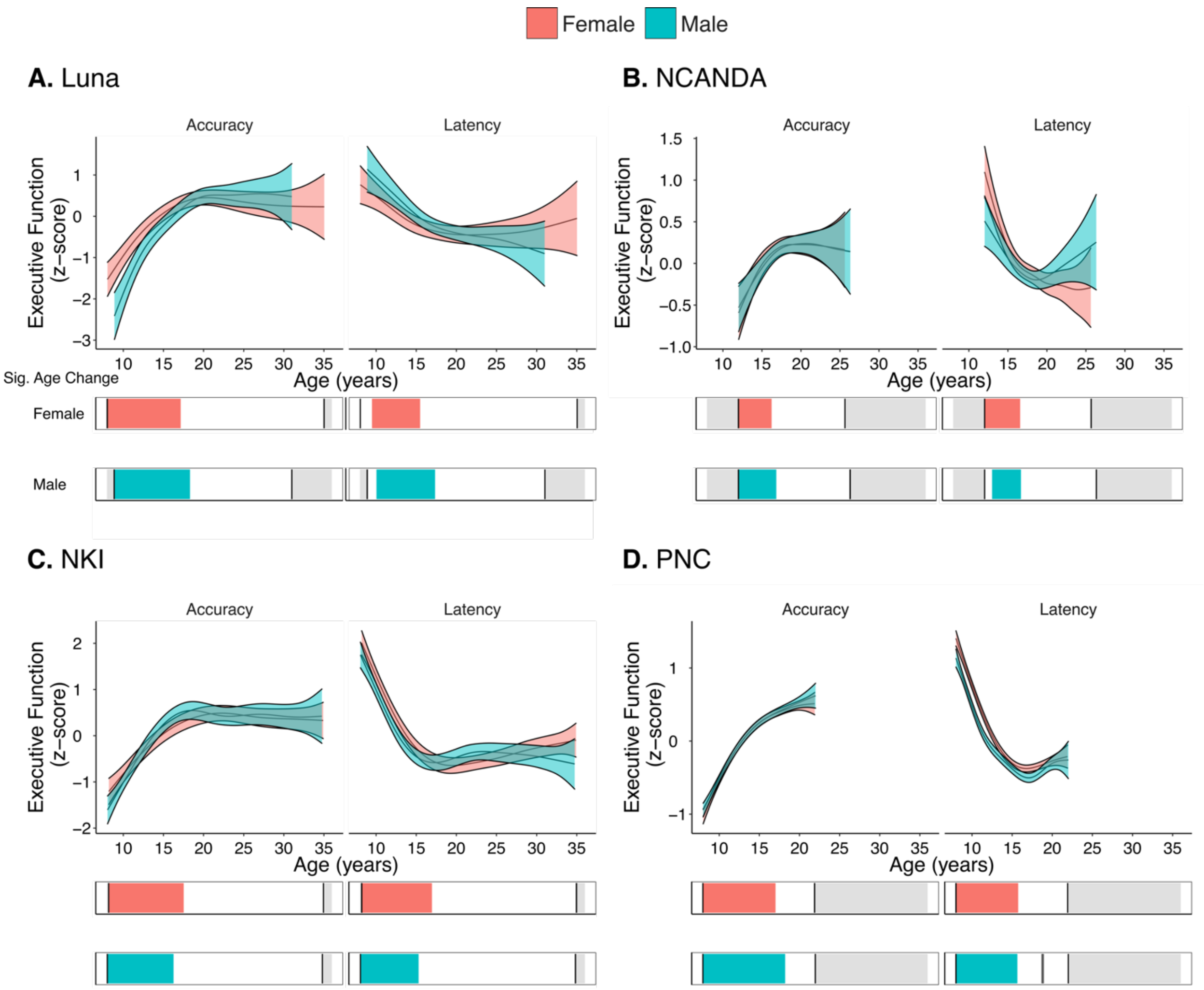

Line plots display fitted age trajectories via GAM/GAMM models of domain general accuracy and latency executive function measures (equally weighted composite metrics, z score sum of all accuracy, latency measures) for males and females for Luna (**A**), NCANDA (**B**), NKI (**C**), and PNC **(D)** datasets. All measures scaled to per-dataset standard deviation (z) units. Bars below line plots display color (female: red; male: blue) if significant ($p < .05$ [two-sided] via simultaneous confidence intervals from posterior simulation of first derivative of GAM/GAMM fits; see Methods) age-related change is observed at that age, with the same procedures as used in Main Text Figure 2. Color scale is matched across measures and datasets and indicates expected direction of each plot (e.g., accuracy increases shown as red for females, blue for males; latency decreases shown as red for females, blue for males). On the rare occurrence where a significant change was observed in the opposite direction of the expected direction (PNC for males on latency) this color is set to black. As in other similar panels (Figure 3 Main Text), grey sections in bar plots indicate no data available for that dataset. Males and females were similar in both the magnitude and timing of executive function development across datasets and any small differences that were observed did not replicate across samples.

Supplementary Figure S14. The Magnitude and Timing of Executive Function Development is Consistent with Primary Results when Statistically Covarying for Parental Education

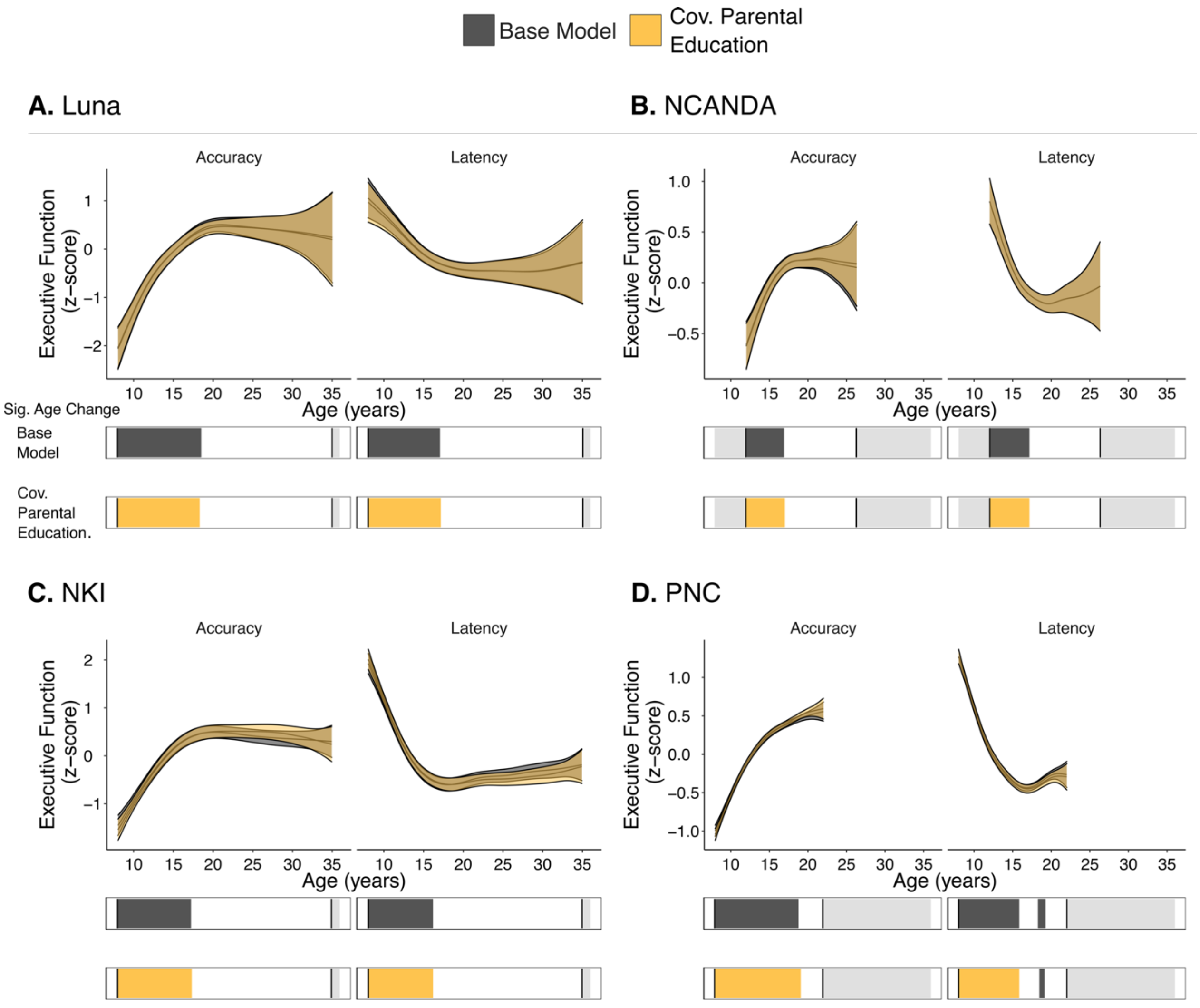

Line plots display fitted age trajectories of domain general accuracy and latency executive function measures (equally weighted composite metrics, z score sum of all accuracy, latency measures) for base GAM/GAMM models (dark grey; i.e., those presented in Main text analysis; only covariate is visit number for longitudinal models [Luna, NCANDA], no covariates for cross-sectional models [NKI, PNC]) and models that additionally covaried for parental education (yellow; smoothed effect in GAM/GAMM models of mean of guardian 1 (e.g., maternal) and guardian 2 (e.g., paternal) years of education) for Luna (**A**), NCANDA (**B**), NKI (**C**), and PNC (**D**). All measures scaled to per-dataset standard deviation (z) units. Bars below line plots display color (base model: dark grey; covarying parental education: yellow) if significant ($p < .05$ [two-sided] via simultaneous confidence intervals from posterior simulation of first derivative of GAM/GAMM fits; see Methods) age-related change is observed at that age, with the same procedures as used in Main Text Figure 2. Color scale is matched across measures and datasets and indicates expected direction of each plot. On the rare occurrence where a significant change was observed in the opposite direction of the expected direction (PNC for latency) this color is set to grey. As in other similar panels (Figure 3 Main Text), grey sections in bar plots indicate no data available for that dataset. The magnitude and timing of executive function development were nearly identical when covarying for parental education.

Supplementary Figure S15. The Magnitude and Timing of Executive Function Development is Consistent with Primary Results when Statistically Covarying for Family Income

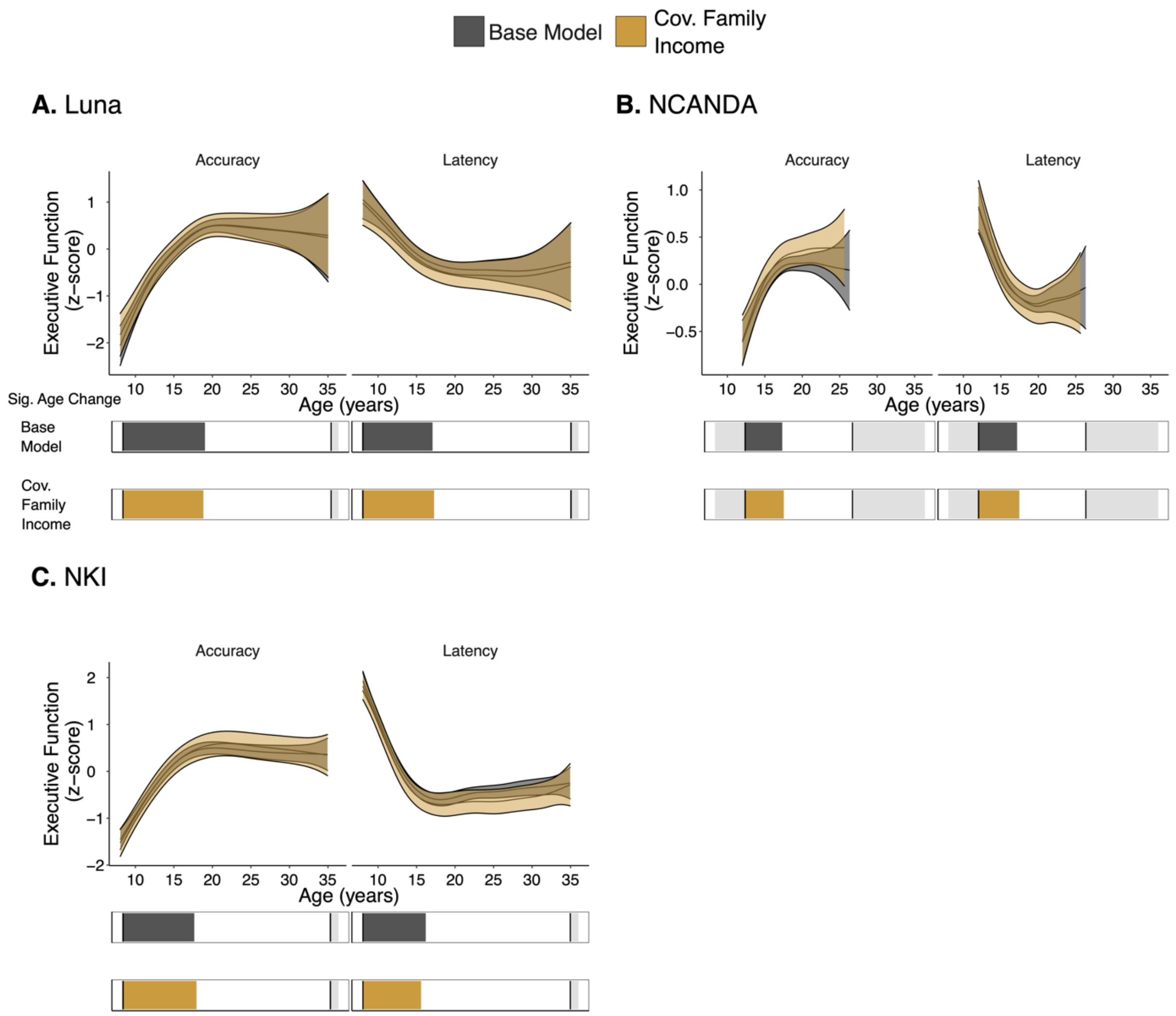

Line plots display fitted age trajectories of domain general accuracy and latency executive function measures (equally weighted composite metrics, z score sum of all accuracy, latency measures) for base GAM/GAMM models (dark grey; i.e., those presented in Main text analysis; only covariate is visit number for longitudinal models [Luna], no covariates for cross-sectional models [NKI, PNC]) and models that additionally covaried for family income (dark yellow; family income coded as in Supplementary Table S1 with covariate model set to level 75-99K) for Luna (**A**), NCANDA (**B**), NKI (**C**). All measures scaled to per-dataset standard deviation (z) units. Bars below line plots display color (base model: dark grey; covarying family income: dark yellow) if significant ($p < .05$ [two-sided] via simultaneous confidence intervals from posterior simulation of first derivative of GAM/GAMM fits; see Methods) age-related change is observed at that age, with the same procedures as used in Main Text Figure 2. Color scale is matched across measures and datasets and indicates expected direction of each plot. As in other similar panels (Figure 3 Main Text), grey sections in bar plots indicate no data available for that dataset. Subtle differences in the magnitude of executive function accuracy, but not the timing, were observed when covarying for family income in NCANDA, compared to the base model. However, these differences did not replicate across samples, as the magnitude and timing of executive function development were nearly identical for Luna and NKI datasets when covarying for family income.

Supplementary Figure S16. The Magnitude and Timing of Executive Function Development is Consistent with Primary Results when Statistically Covarying for Culturally Acquired Knowledge.

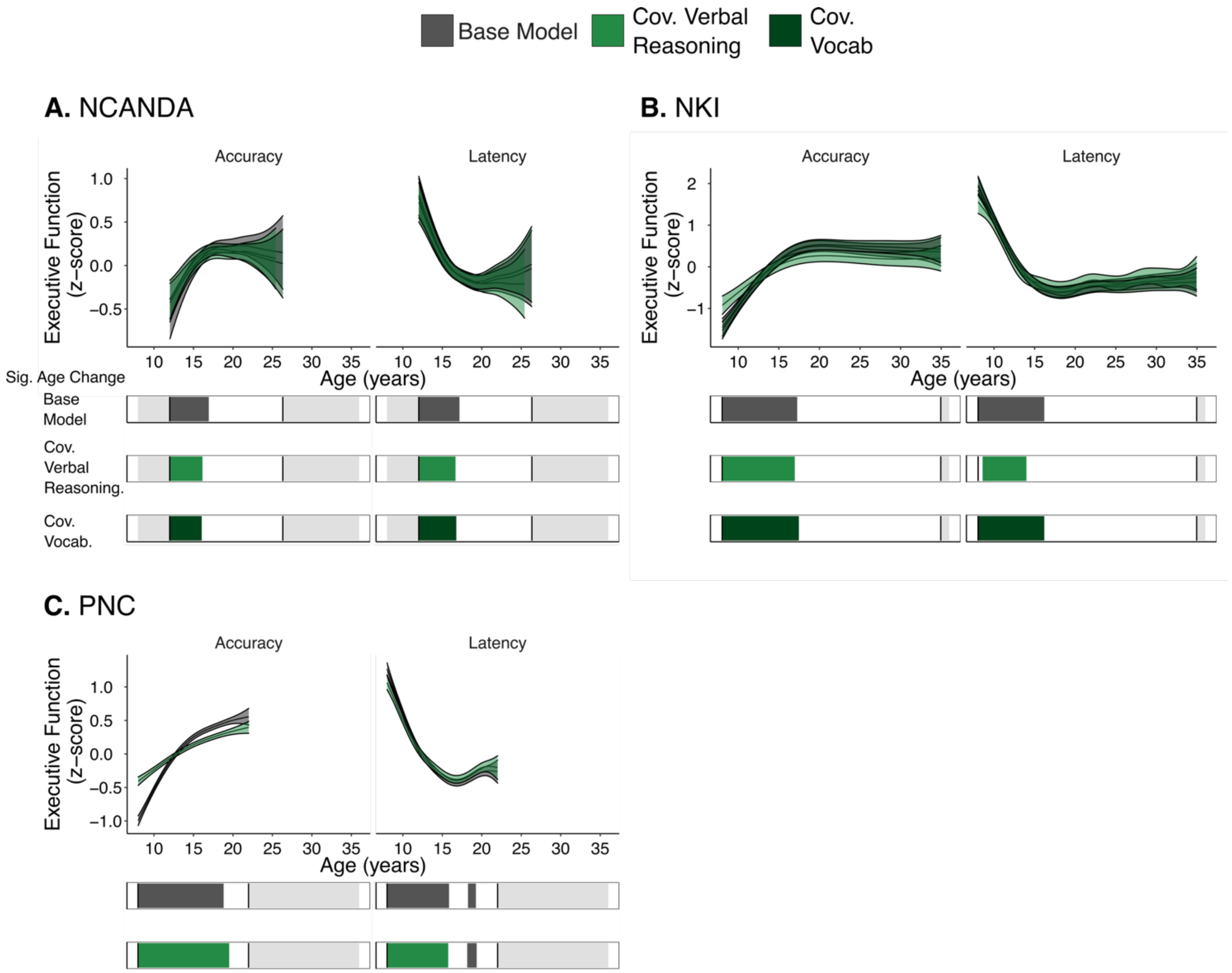

Line plots display fitted age trajectories of domain general accuracy and latency executive function measures (equally weighted composite metrics, z score sum of all accuracy, latency measures) for base GAM/GAMM models (dark grey; i.e., those presented in Main text analysis; only covariate is visit number for longitudinal models [NCANDA], no covariates for cross-sectional models [NKI, PNC]) and models that additionally covaried (smoothed effect in GAM/GAMM models) for verbal reasoning (light green; NCANDA, NKI, PNC: Penn CNP Verbal Reasoning Test [number of correct responses]) or vocabulary (dark green; NCANDA: Penn Vocabulary Test Correct Response [number of correct responses]; NKI WASI-II Vocabulary [T score]) for NCANDA (**A**), NKI (**B**), and PNC (**C**). All measures scaled to per-dataset standard deviation (z) units. Bars below line plots display color (base model: dark grey; covarying verbal reasoning: light green; covarying vocabulary: dark green) if significant ($p < .05$ [two-sided] via simultaneous confidence intervals from posterior simulation of first derivative of GAM/GAMM fits; see Methods) age-related change is observed at that age, with the same procedures as used in Main Text Figure 2. Color scale is matched across measures and datasets and indicates expected direction of each plot. On the rare occurrence where a significant change was observed in the opposite direction of the expected direction (PNC for latency) this color is set to grey. As in other similar panels (Figure 3 Main Text), grey sections in bar plots indicate no data available for that dataset. The magnitude and timing of executive function development were nearly identical when covarying for these measures of culturally acquired knowledge and any small differences that were observed did not replicate across samples.

Supplementary Figure S17. The Magnitude and Timing of Executive Function Development is Consistent when Excluding Participants with a Current, Primary Psychiatric Condition

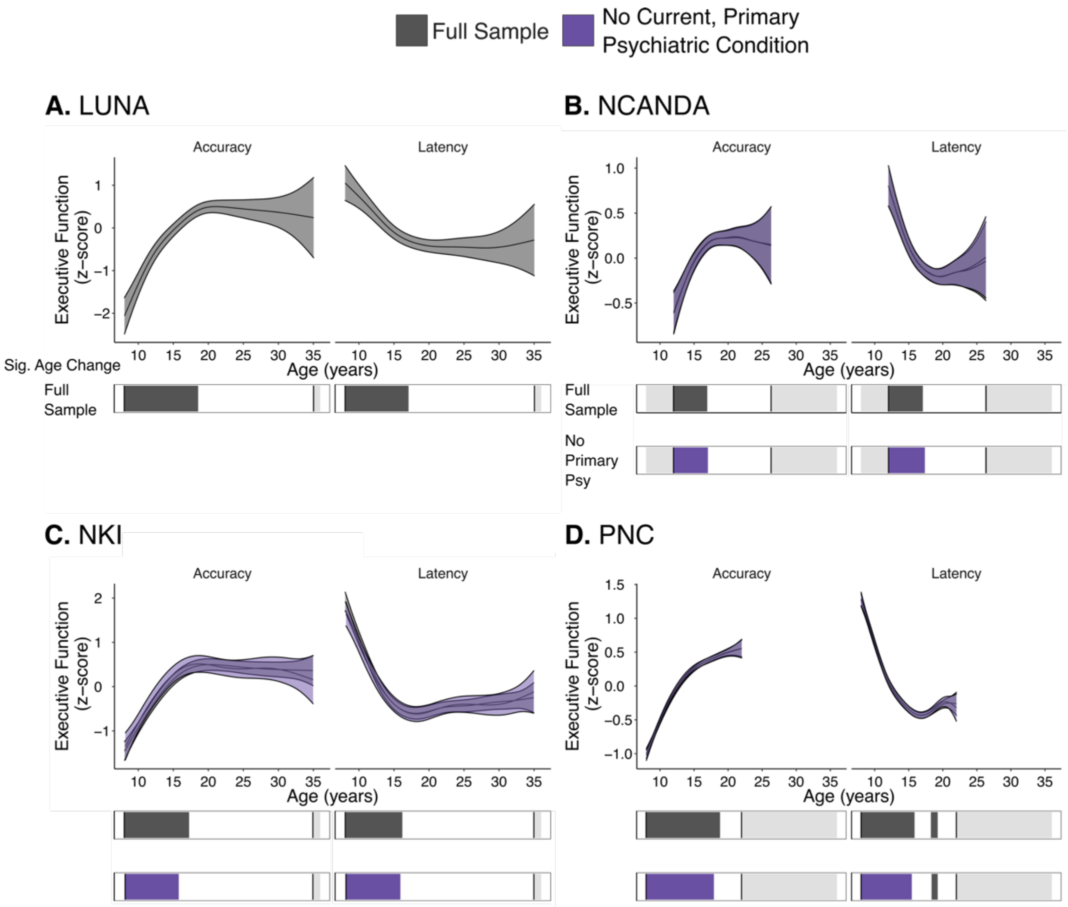

Line plots display fitted age trajectories via GAM/GAMM models of domain general accuracy and latency executive function measures (equally weighted composite metrics, z score sum of all accuracy, latency measures) for the full sample (dark grey; i.e., those presented in Main text analysis; Luna (**A**), NCANDA (**B**), NKI (**C**), PNC (**D**)) and the subsample of participants that did not meet the per-sample criteria for having a current, primary psychiatric condition (purple; **B**, **C**, **D;** see Supplementary Methods). The Luna sample had primary psychiatric diagnosis as an exclusion (see Methods) and thus are presented here only as full sample. All measures scaled to per-dataset standard deviation (z) units. Bars below line plots display color (full sample: dark grey; excluding participants with current, primary psychiatric condition: purple) if significant ($p < .05$ [two-sided] via simultaneous confidence intervals from posterior simulation of first derivative of GAM/GAMM fits; see Methods) age-related change is observed at that age, with the same procedures as used in Main Text Figure 2. Color scale is matched across measures and datasets and indicates expected direction of each plot. On the rare occurrence where a significant change was observed in the opposite direction of the expected direction (PNC for latency) this color is set to grey. As in other similar panels (Figure 3 Main Text), grey sections in bar plots indicate no data available for that dataset. The magnitude and timing of executive function development were nearly identical to the full sample when excluding participants with current, primary psychiatric condition and these insights are further strengthened by replication used throughout the manuscript from the Luna sample that had a psychiatric diagnosis (either in the participant or first-degree relative) as an exclusion criterion.

Supplementary Tables

Supplementary Table S1. Baseline Sociodemographic Characteristics of Included Datasets and the American Community Survey

	Dataset	Luna	NCANDA	NKI	PNC	ACS 2011-2015
Characteristic		%	%	%	%	%
Race/Ethnicity	NH White	78.7	64.1	53.3	56.2	59.8
	NH Black/AA	12.2	11.9	19.0	35.6	11.7
	Asian, AIAN, NHPI	3.2	8.2	9.1	0.4	7.0
	Hispanic	5.9	11.6	16.9	5.8	12.3
	Other	—	4.3	1.7	2.0	9.3
Sex	Male	47.4	49.1	51.5	47.9	50.9
	Female	52.6	50.9	48.5	52.1	49.1
Family Income	<25k	0.0	6.0	16.0	—	24.6
	25k-49k	8.3	11.3	19.8	—	22.2
	50k-74k	13.5	12.9	18.6	—	17.1
	75k-99k	21.2	13.5	12.9	—	12.1
	100k-199k	35.3	34.5	24.8	—	18.8
	200k+	21.7	21.9	8.1	—	5.3
Guardian Highest Education	Incomplete High School	< 1	2.7	4.5	4.4	—
	High School	14.2	6.0	22.0	27.1	—
	1-3 Years of College	29.5	16.1	23.8	24.9	—
	Bachelor's	31.3	35.7	27.8	26.9	—
	Postgraduate	23.3	39.6	21.9	16.5	—

Note. Characteristic coding for race/ethnicity, sex, and family income based on recent, large-scale multi-site developmental approach[97]. Non-Hispanic (NH). African American (AA). American Indian and Alaska Native (AIAN). Native Hawaiian and Pacific Islander (NHPI). Data unavailable/not collected (—). American Community Survey (ACS) displays population estimates of the United States. Guardian Highest Education based on explicit identification of given categories (Luna) or approximation based on years of reported education (incomplete High School < 12, High School = 12, 1-3 Years of College = 13-15, Bachelor's = 16, Postgraduate > 16). All variables for primary datasets were assessed via self-report. See Methods for information on recruitment. See Supplementary Figure S1 for age distributions of participants by study visit.

Supplementary Table S2. Executive Function Measures by Dataset

Dataset	Subdomain(s)	Executive Function Measures
Luna	Inhibition	Antisaccade (ANTI), Fixation Breaks (FIX)
	Inhibition/ Switching	Mixed Antisaccade (MIX)
	Planning	Stockings of Cambridge (SOC)
	Working Memory	Memory Guided Saccade (MGS), Delayed Matching to Sample (DMS), Spatial Span (SSP)
NCANDA	Attention/ Inhibition	Penn Continuous Performance Test (PCPT)
	Inhibition	Stroop (STRP)
	Sorting	Penn Conditional Exclusion Test (PCET)
	Working Memory	Penn N-Back Test (PNBK)
NKI	Attention/ Inhibition	Penn Continuous Performance Test (PCPT),
	Inhibition	D-KEFS Color Word Interference (CWI), D-KEFS Trails (TMT)
	Planning	D-EKFS Tower (TOW), D-KEFS Design Fluency (DFL)
	Sorting	Penn Conditional Exclusion Test (PCET)
	Working Memory	Penn N-Back Test (PNBK)
PNC	Attention/ Inhibition	Penn Continuous Performance Test (PCPT)
	Sorting	Penn Conditional Exclusion Test (PCET)
	Working Memory	Penn N-Back Test (PNBK)

Note, See Methods for individual measure and scoring and Supplementary Table 3 for dataset variable names. Subdomain(s) were based on author consensus and with respect to test descriptions and materials.

Supplementary Table S3. GAM/GAMM Statistics from Full Age Models, Dataset Variable Names

Dataset	Var Label	Var Type	Variable Name	Effective *df*	*F*	Adjusted P Value
Luna	ANTI	lat	Anti_CRLat	4.41	12.57	0.000
Luna	ANTI	acc	Anti_CRR	5.08	30.04	0.000
Luna	COMP	acc	Accuracycomposite	5.40	32.22	0.000
Luna	COMP	lat	Latencycomposite	4.23	16.32	0.000
Luna	DMS	lat	DMS.Median.correct.latency	2.75	12.18	0.000
Luna	DMS	acc	DMS.PC	2.94	10.02	0.000
Luna	FIX	acc	nfixbreak_fl	2.88	5.16	0.004
Luna	MGS	acc	best_acc_m_exclude_fl	3.00	7.58	0.000
Luna	MGS	lat	first_lat_m_exclude	4.29	18.26	0.000
Luna	MIX	lat	Mix_CRLat	4.57	9.89	0.000
Luna	MIX	acc	Mix_CRR	3.99	19.41	0.000
Luna	SOC	lat	SOC.Overallmeaninitialthinkingtime	1.97	1.83	0.145
Luna	SOC	acc	SOC.Problems.solved.in.minimum.moves	3.73	17.00	0.000
Luna	SSP	acc	SSP.Span.length	4.54	16.16	0.000
NCANDA	COMP	acc	Accuracycomposite	4.32	15.17	0.000
NCANDA	COMP	lat	Latencycomposite	4.87	23.57	0.000
NCANDA	PCET	acc	cnp_pcet_pcet_acc2	1.96	1.71	0.175
NCANDA	PCET	lat	cnp_pcet_pcetrtcr	2.84	5.82	0.001
NCANDA	PCTP	acc	cnp_spcptnl_scpt_tp	5.36	29.89	0.000
NCANDA	PCTP	lat	cnp_spcptnl_scpt_tprt	4.56	19.49	0.000
NCANDA	PNBK	acc	cnp_sfnb2_sfnb_mcr	1.70	1.51	0.130
NCANDA	PNBK	lat	cnp_sfnb2_sfnb_mrtc	3.07	5.59	0.001
NCANDA	STRP	lat	stroop_total_mean	5.19	20.70	0.000
NKI	COMP	acc	Accuracycomposite	3.98	69.93	0.000
NKI	COMP	lat	Latencycomposite	5.23	103.69	0.000
NKI	CWI	lat	CWIlat	4.86	108.63	0.000
NKI	DFL	acc	DFLacc	3.85	54.14	0.000
NKI	PCET	acc	PCET_PCET_ACC2	3.60	11.99	0.000
NKI	PCET	lat	PCET_PCETRTCR	4.65	15.75	0.000
NKI	PCTP	acc	SPCPTNL_SCPT_TP	3.13	43.16	0.000
NKI	PCTP	lat	SPCPTNL_SCPT_TPRT	7.56	39.05	0.000
NKI	PNBK	acc	SLNB2_SLNB_MCR	3.21	17.97	0.000
NKI	PNBK	lat	SLNB2_SLNB_MRTC	4.82	22.90	0.000
NKI	TMT	lat	TMTlat	4.84	55.86	0.000
NKI	TOW	acc	TOWacc	4.73	13.74	0.000
PNC	COMP	acc	Accuracycomposite	3.86	467.39	0.000
PNC	COMP	lat	Latencycomposite	6.22	405.47	0.000
PNC	PCET	acc	PCET_ACC2	4.29	106.59	0.000
PNC	PCET	lat	PCET_RTCR	3.40	45.71	0.000
PNC	PCTP	acc	PCPT_T_TP	3.29	399.10	0.000
PNC	PCTP	lat	PCPT_T_TPRT	4.77	973.05	0.000
PNC	PNBK	acc	LNB_MCR	3.70	262.57	0.000
PNC	PNBK	lat	LNB_MRTC	5.73	198.16	0.000

Note, Statistics from GAM/GAMM Models Presented in Figure 1. Var label is the task abbreviation used throughout manuscript (see Table 1). Var type presents whether measure is latency (lat) or accuracy (acc). Variable name presents variable name from original dataset. Effective degrees of freedom (df), F statistic, and Adjusted *P* Value (within-dataset Bonferroni correction) are from smooth effect of age. *P* values are calculated via default procedures of GAM that perform an equality test of all parameters of the smoothed term to zero. See Methods for additional detail on model specification. See Main Text Figure 2 for testing local, age-specific effects at varying developmental periods.

Supplementary Table S4. Correlation (Linear, Bivariate) Matrices for All Datasets

Linear, bivariate correlation matrices for all datasets. For longitudinal datasets (Luna, NCANDA), baseline refers to the first visit, longitudinal refers to within-person correlation (via disaggregation, see Methods).

Luna Baseline

ANTI_acc											
MIX_acc	0.786										
DMS_acc	0.273	0.289									
SSP_acc	0.312	0.27	0.372								
FIX_acc	0.401	0.33	0.233	0.148							
SOC_acc	0.329	0.368	0.389	0.341	0.285						
MGS_acc	0.327	0.272	0.172	0.229	0.163	0.268					
ANTI_lat	-0.071	-0.097	-0.143	-0.307	-0.02	-0.177	-0.129				
MIX_lat	-0.171	-0.124	-0.158	-0.32	-0.07	-0.129	-0.204	0.743			
SOC_lat	0.097	0.157	0.152	0.069	0.174	0.4	0.099	-0.038	-0.025		
DMS_lat	-0.187	-0.267	-0.046	-0.225	-0.141	-0.039	-0.023	0.184	0.177	0.076	
MGS_lat	-0.377	-0.362	-0.191	-0.385	-0.099	-0.346	-0.196	0.438	0.356	-0.067	0.057

Luna Longitudinal

ANTI_acc											
MIX_acc	0.521										
DMS_acc	0.206	0.123									
SSP_acc	0.239	0.112	0.189								
FIX_acc	0.271	0.138	0.113	0.148							
SOC_acc	0.189	0.138	0.103	0.099	0.093						
MGS_acc	0.184	0.09	0.096	0.098	0.145	0.072					
ANTI_lat	-0.298	-0.182	-0.17	-0.274	-0.138	-0.362	-0.172				
MIX_lat	-0.289	-0.159	-0.069	-0.257	-0.059	-0.219	-0.141	0.708			
SOC_lat	-0.085	-0.016	-0.034	-0.14	-0.051	0.143	-0.032	0.043	0.059		
DMS_lat	-0.243	-0.167	0.022	-0.115	-0.04	-0.092	-0.051	0.299	0.207	0.115	
MGS_lat	-0.311	-0.207	-0.193	-0.215	-0.117	-0.189	-0.213	0.474	0.362	-0.021	0.108

NCANDA Baseline

PCET_acc						
PNBK_acc	0.154					
PCTP_acc	0.142	0.221				
PCET_lat	-0.23	-0.177	-0.119			
PNBK_lat	-0.042	-0.171	-0.025	0.079		
PCTP_lat	-0.006	-0.091	-0.144	0.101	0.449	
STRP_lat	-0.059	-0.197	-0.219	0.22	0.323	0.358

NCANDA Longitudinal

PCET_acc						
PNBK_acc	0.102					
PCTP_acc	0.157	0.144				
PCET_lat	-0.306	-0.085	-0.133			
PNBK_lat	0.001	-0.084	-0.04	0.026		
PCTP_lat	0.054	-0.025	-0.047	0.026	0.299	
STRP_lat	-0.134	-0.113	-0.265	0.171	0.167	0.086

NKI

PCET_acc									
PNBK_acc	0.307								
PCTP_acc	0.239	0.219							
TOW_acc	0.311	0.166	0.129						
DFL_acc	0.344	0.316	0.327	0.39					
PCET_lat	-0.286	-0.221	-0.171	-0.193	-0.327				
PNBK_lat	-0.205	-0.324	-0.123	-0.187	-0.307	0.279			
PCTP_lat	-0.167	-0.245	-0.359	-0.178	-0.399	0.254	0.495		
CWI_lat	-0.36	-0.387	-0.388	-0.344	-0.625	0.367	0.483	0.51	
TMT_lat	-0.412	-0.406	-0.392	-0.366	-0.597	0.422	0.409	0.471	0.677

PNC

PCET_acc					
PCTP_acc	0.185				
LNB_acc	0.29	0.312			
PCET_lat	-0.21	-0.131	-0.193		
PCTP_lat	-0.178	-0.303	-0.296	0.162	
LNB_lat	-0.133	-0.142	-0.257	0.174	0.548
