# Supplementary material for: A canonical trajectory of executive function maturation from adolescence to adulthood
Source: Nat Commun. 2023 Oct 30;14:6922. doi: 10.1038/s41467-023-42540-8 (PMC10616171; doi:10.1038/s41467-023-42540-8)
Supplement: Supplementary file 3 — Reporting Summary [file 41467_2023_42540_MOESM3_ESM.pdf]

Corresponding author(s): Brenden Tervo-Clemmens
Last updated by author(s): Aug 29, 2023

Reporting Summary

Nature Portfolio wishes to improve the reproducibility of the work that we publish. This form provides structure for consistency and transparency in reporting. For further information on Nature Portfolio policies, see our Editorial Policies and the Editorial Policy Checklist.

Statistics

For all statistical analyses, confirm that the following items are present in the figure legend, table legend, main text, or Methods section.

n/a	Confirmed	
☐	☒	The exact sample size (*n*) for each experimental group/condition, given as a discrete number and unit of measurement
☐	☒	A statement on whether measurements were taken from distinct samples or whether the same sample was measured repeatedly
☐	☒	The statistical test(s) used AND whether they are one- or two-sided<br>*Only common tests should be described solely by name; describe more complex techniques in the Methods section.*
☐	☒	A description of all covariates tested
☐	☒	A description of any assumptions or corrections, such as tests of normality and adjustment for multiple comparisons
☐	☒	A full description of the statistical parameters including central tendency (e.g. means) or other basic estimates (e.g. regression coefficient) AND variation (e.g. standard deviation) or associated estimates of uncertainty (e.g. confidence intervals)
☐	☒	For null hypothesis testing, the test statistic (e.g. *F*, *t*, *r*) with confidence intervals, effect sizes, degrees of freedom and *P* value noted<br>*Give P values as exact values whenever suitable.*
☒	☐	For Bayesian analysis, information on the choice of priors and Markov chain Monte Carlo settings
☐	☒	For hierarchical and complex designs, identification of the appropriate level for tests and full reporting of outcomes
☐	☒	Estimates of effect sizes (e.g. Cohen's *d*, Pearson's *r*), indicating how they were calculated

Our web collection on statistics for biologists contains articles on many of the points above.

Software and code

Policy information about availability of computer code

Data collection	No software was used for data collection.
Data analysis	Data were analyzed using R version 4.1.2 (2021), with psych (version 2.1.9), mgcv (version 1.8-38), gratia (version 0.7.0), metafor (3.0-2), nFactors (version 2.4.1), and performance (version 0.8.0) packages.<br><br>Analysis code for the current project and summary data of the canonical executive function trajectory are available at https://github.com/tervoclemmensb/Executive Function_ Charting

For manuscripts utilizing custom algorithms or software that are central to the research but not yet described in published literature, software must be made available to editors and reviewers. We strongly encourage code deposition in a community repository (e.g. GitHub). See the Nature Portfolio guidelines for submitting code & software for further information.

Data

Policy information about availability of data

All manuscripts must include a data availability statement. This statement should provide the following information, where applicable:

- Accession codes, unique identifiers, or web links for publicly available datasets
- A description of any restrictions on data availability
- For clinical datasets or third party data, please ensure that the statement adheres to our policy

Deidentified data for all datasets used in this project are available in public repositories pending appropriate data use agreements.
Luna sample: https://nda.nih.gov/edit_collection.html?id=2831
NCANDA: ncanda.org (Release 4Y V02)
NKI: fcon_1000.projects.nitrc.org/indi/enhanced/
PNC: ncbi.nlm.nih.gov/projects/gap/cgi-bin/study.cgi?study_id=phs000607.v3.p2

Research involving human participants, their data, or biological material

Policy information about studies with human participants or human data. See also policy information about sex, gender (identity/presentation), and sexual orientation and race, ethnicity and racism.

Reporting on sex and gender	Differences according to biological sex are reported and explored in Supplemental Figure S13
Reporting on race, ethnicity, or other socially relevant groupings	We report population factors when describing the sample and terminology from related national survey and epidemiological datasets and provide references therein. "Studies relied on community-based samples from across the United States (see Methods) that were balanced for biological sex at birth and in the aggregate, met national patterns of race and ethnicity (Supplemental S1). Family income varied both within and between datasets, but as in previous reports across behavioral sciences, was generally higher than national averages (Supplemental S1)." We report these sample characteristics in Supplemental Table 1.
Population characteristics	Publicly available data were analyzed, with original studies drawing from community-based samples of children and adolescents (ages 8-35-years-old; approximately balanced between males and females) from multiple locations across the United States (Pittsburgh PA, Durham NC, Portland OR, Menlo Park CA, San Diego CA, Rockland County NY, Philadelphia PA). We report broader sample characteristics and those specific to individual datasets in full in Supplemental Table 1.
Recruitment	As above, we detail in the manuscript that the data here are from community-based samples.
Ethics oversight	In all four datasets, research protocols were approved by the relevant institutional review boards (Luna Dataset: University of Pittsburgh; NCANDA: Duke University, Oregon Health and Sciences University, SRI International, University of Pittsburgh, University of California San Diego; NKI: Nathan Kline Institute; PNC: The University of Pennsylvania and Children's Hospital of Philadelphia)

Note that full information on the approval of the study protocol must also be provided in the manuscript.

Field-specific reporting

Please select the one below that is the best fit for your research. If you are not sure, read the appropriate sections before making your selection.

☐ Life sciences ☒ Behavioural & social sciences ☐ Ecological, evolutionary & environmental sciences

For a reference copy of the document with all sections, see nature.com/documents/nr-reporting-summary-flat.pdf

Behavioural & social sciences study design

All studies must disclose on these points even when the disclosure is negative.

Study description	Four independent datasets (two cross-sectional, two longitudinal) were used to assess quantitative measures of executive function, participant age, and relevant sociodemographic factors.
Research sample	Data for this project were provided from participants of four existing projects (all with publicly available data). One internal dataset (Luna Dataset) and three external datasets (National Consortium on Alcohol & Neurodevelopment in Adolescence [NCANDA], Nathan Kline Institute-Rockland Sample [NKI], Philadelphia Neurodevelopmental Cohort [PNC]) were included based on 1) their inclusion of executive function tasks performed in a developmental or lifespan dataset spanning the entirety of the adolescent period and 2) to aggregate the largest possible dataset to explore the aims of this project. The primary focus of the current work was on the adolescent period. To explicitly capture transitions into and out of adolescence as well as the entire adolescent period, we included participants ranging from late childhood to adulthood (8-35-years old). Lower (8-years-old) and upper (35-years-old) age ranges were selected to be as inclusive as possible, given the overarching goal of capturing non-linear developmental trajectories, while also ensuring that at least two separate datasets had participants in each age range.

Participants for each existing dataset were recruited from the community/ broader catchment areas of the prior studies (Pittsburgh PA, Durham NC, Portland OR, Menlo Park CA, San Diego CA, Rockland County NY, Philadelphia PA). Studies relied on convenience-based community sampling that did not adhere or target strict population representativeness, nor did the original datasets include sampling weights to pursue these types of population estimates. These issues are explored and further detailed in the Supplementary Material (Supplemental Methods, Supplemental Figure S2).

Sampling strategy

Existing datasets, with convenience sampling from the community, were used in this analysis and are detailed above and in original protocol papers.
Luna, B., Garver, K. E., Urban, T. A., Lazar, N. A., & Sweeney, J. A. (2004). Maturation of cognitive processes from late childhood to adulthood. Child development, 75(5), 1357-1372.
Nooner, K. B. et al. The NKI-Rockland sample: a model for accelerating the pace of discovery science in psychiatry. Frontiers in neuroscience 6, 152 (2012).
Brown, S. A. et al. The National Consortium on Alcohol and NeuroDevelopment in Adolescence (NCANDA): a multisite study of adolescent development and substance use. Journal of studies on alcohol and drugs 76, 895–908 (2015).
Calkins, M. E. et al. The Philadelphia Neurodevelopmental Cohort: constructing a deep phenotyping collaborative. Journal of Child Psychology and Psychiatry 56, 1356–1369 (2015).

Data collection

Data from Luna, NCANDA, NKI, and PNC datasets were used in the current project based on their inclusion of executive function tasks performed in a developmental or lifespan dataset that spanned the adolescent period. Classification of "executive function" tasks was based on prior theoretical5 and empirical work15,34,42,78, with a general operationalization of "goal-directed" cognitive behaviors that encompassed processes of "inhibition", "attention", "working memory", "switching", or "planning". Where possible, prior work with the included tasks and datasets and/or test authors34 was used to define whether specific tasks indexed "executive function". To avoid potential influences of verbal skills potentially related to educational attainment, measures relying heavily on reading and language skills were not included (e.g., DKEFS-Twenty Questions, DKEFS-Proverb Test) as primary executive function assessments, but the influence of culturally acquired knowledge was shown to not influence primary results in a sensitivity analysis (Supplemental Figure S7). Wherever possible, both accuracy and latency measures were selected, except when precedence from research or clinical assessment was clear on a predominant use of accuracy (e.g., DKEFS Color-Word Interference, Design Fluency, Sorting, and Tower) or latency (e.g., DKEFS Trail Making Test) measures owing to nearly universal ceiling/floor performance of the corresponding accuracy/latency measure and/or the corresponding measure was not collected/available. See Table 1 for the conceptualized subdomains of the included executive function tasks based on author consensus and original test descriptions. See Supplemental Table S2 for reproducible variable names for public datasets (NCANDA, NKI, PNC).
Based on the above criteria, the Luna dataset included twelve measures from six executive function tasks that were completed at each visit: Antisaccade (ANTI), Memory Guided Saccade (MGS), a "Mixed" (MIX) Antisaccade/Visually Guided Saccade/Fixation task, Cambridge Neuropsychological Test Automated Battery [CANTAB] Delayed Matching to Sample (DMS), CANTAB Spatial Span (SSP), CANTAB Stockings of Cambridge (SOC). Each of these tasks have been described in detail elsewhere (see for example 15,44). Scoring procedures and outcome measures were based on previous work from our group and general use in the literature. Briefly, the Antisaccade task required participants to inhibit a proponent response (saccade) to a peripheral stimulus (in four possible locations along the horizontal meridian) and saccade towards the opposite hemifield. Both accuracy (correct response rate across trials) and latency (median speed of antisaccades on correct trials) of the Antisaccade task were examined. A second "Mixed" version of the Antisaccade task was also performed, where participants performed an antisaccade but trials with different task demands were also interleaved. Specifically, in 1/3rd of trials, participants were required to saccade towards the peripheral stimulus (visually guided saccade) or in 1/3rd number of trials, simply maintain fixation. Both accuracy and latency of this mixed version were examined, but only calculated for the antisaccade trials (with the same scoring procedure as above), given the visually guided saccade is not thought to rely on executive function (see 15) and the number of fixation errors was included in a different measure that captured this performance in a goal-oriented context (see below). The Memory Guided Saccade task required participants to saccade towards a peripheral stimulus (in four possible locations along the horizontal meridian), remember its location during a subsequent fixation period, and then saccade towards the remembered location when no stimulus was presented. Both accuracy (difference in degrees between initial saccade and the most precise saccade the final phase79, when no stimulus was presented) and latency (median speed of the initial saccade during the final phase across trials79) of the Memory Guided Saccade task were examined. We also calculated the number of fixation breaks (FIX) during the middle phase of the memory guided saccade task as a putative measure of inhibition. In addition to the three eye movement tasks, the Luna dataset also included the Delayed Matching to Sample, Spatial Span, and Stockings of Cambridge tasks from the CANTAB Battery, each of which have been broadly used and whose stimuli can be found online (see www.cambridgecognition.com/cantab/). Standard accuracy (Delayed Matching to Sample: Percent Correct; Spatial Span: Span Length; Stockings of Cambridge: Problems Solved in Minimum Moves) and latency (Delayed Matching to Sampe: Median Correct Latency; Stockings of Cambridge: Mean Initial Thinking Time) measures from each of the three CANTAB tasks were examined. For interpretive consistency across measures in the Luna dataset, the direction of the scoring of two accuracy measures (Memory Guided Saccade "inaccuracy" [see above]; Number of Fixation Breaks) was multiplied by -1 to ensure that higher scores indexed better performance on all accuracy measures.
The NCANDA, PNC, and NKI datasets used versions of the University of Pennsylvania Computerized Neurocognitive Battery (CNB; https://webcnp.med.upenn.edu/). The current project utilized data from three CNB tasks that met our operationalization of executive function and have been classified as "executive" by the CNB authors34, the Penn Conditional Exclusion Test (PCET), a Penn N-Back Test (PNBK; NCANDA: Penn Short Fractal N-back Test [PNB-F]; PNC & NKI: Penn Letter N-Back Test [PNB-L]), and the Penn Continuous Performance Test: Number and Letter version (PCPT). Standard outcome measures for each task were included for accuracy (PCET: calculated accuracy measure ("PCET ACC2"); PNB-F: true positive [correct] responses for 1-back and 2-back trials; PNB-L: true positive [correct] responses for 1-back and 2-back trials; PCPT: sum of true positives for number and letter trials) and latency (PCET: median response time for correct responses; PNB-F: mean of median response time for 1-back and 2-back trials; PNB-L: mean of median responses for 1-back and 2-back trials, PCPT: median response time for correct response to number trials and letter trials). The NCANDA dataset also included a standard Stroop Test (STRP), where the primary measure of average latency over all correct trials was included. The NKI dataset also included four executive function tasks from the Delis-Kaplan Executive Function System43 (D-KEFS) that were included in the current study: color-word interference (CWI), design fluency (DFL), tower (TOW), and the trail-making test (TMT). Again, standard outcome measures were used for these tasks (CWI latency: average of inhibition and inhibition/switching conditions; correlation amongst these measures: r=.806; DFL Accuracy78,80: switching total correct; TOW: Total Achievement Score Total Raw; TMT : Number-Letter Switching). The DKEFS Sort Task was also available for a small percentage of participant visits within our analytic age range (8-35) for the NKI dataset but was not used because over two thirds of the visits did not have this measure (66.82%), whereas all other NKI measures included had at maximum <4% missingness.

The current researchers were not blind to the study hypotheses or experimental conditions of the original datasets.

Timing	Data in the current project were accessed and analyzed from starting September of 2019 with final analyses completed in May 2022. We note project stoppage from July 2020-July 2021 due to the first author clinical obligations.
Data exclusions	All data processing and statistical analyses were performed in R version 4.1.2 (2021)81. Luna dataset eye-tracking data was scored with the same automatic scoring algorithms from our previous work79,82. Scores for all other tasks were generated through released software from the instrument (e.g., Luna dataset CANTAB) and/or included in official data releases (NCANDA, NKI, PNC datasets).<br>Aggregated data, either from distributed data releases (NCANDA, NKI, PNC) or our in-house database (Luna dataset) were first screened to ensure each visit (participant at testing session) had a valid age, anonymous id variable, and if longitudinal data, visit (i.e., these variables were not missing and were within the expected range, based on the study design) and included expected data. Data that didn't meet these minimum criteria were removed from all analyses. As in our prior work, eye-tracking tasks in the Luna dataset (specific task at specific visit) with more than 30% of trials dropped due to poor eye-tracking or missing (i.e., early session termination; cf., 82) were also removed from all analyses. Next, data inclusion criteria were included to maximize the included dataset sizes and result generalizability, while also ensuring no considerable outlier (i.e., 4 standard deviations and more extreme than 99.9% of the distribution) biased results. Within these procedures, individual executive function measures were first screened for potential univariate leverage points in the association between age and each specific measure within general additive models (GAM: see below) or general additive mixed models (GAMM: see below). Leverage points were defined as those observations (measure for participant at testing session) with a residual from this model that was four standard deviations above the mean and removed from all subsequent analyses. Second, data were examined for potential multivariate outliers among all included executive function measures within each dataset using Mahalanobis distance within the psych package in R75. Sessions (all executive function measures for participant at testing session [i.e., study visit]) with a Mahalanobis distance four standard deviations above the mean were removed from all subsequent analyses.
Non-participation	No new participants were enrolled as part of this study.
Randomization	Data are from observational studies and thus no randomization was performed.

Reporting for specific materials, systems and methods

We require information from authors about some types of materials, experimental systems and methods used in many studies. Here, indicate whether each material, system or method listed is relevant to your study. If you are not sure if a list item applies to your research, read the appropriate section before selecting a response.

Materials & experimental systems

n/a	Involved in the study
☒	☐ Antibodies
☒	☐ Eukaryotic cell lines
☒	☐ Palaeontology and archaeology
☒	☐ Animals and other organisms
☒	☐ Clinical data
☒	☐ Dual use research of concern
☒	☐ Plants

Methods

n/a	Involved in the study
☒	☐ ChIP-seq
☒	☐ Flow cytometry
☒	☐ MRI-based neuroimaging
